# Cell non-autonomous regulation of health and longevity

**DOI:** 10.7554/eLife.62659

**Published:** 2020-12-10

**Authors:** Hillary A Miller, Elizabeth S Dean, Scott D Pletcher, Scott F Leiser

**Affiliations:** 1 Cellular and Molecular Biology Graduate Program, University of MichiganAnn ArborUnited States; 2 Molecular & Integrative Physiology Department, University of MichiganAnn ArborUnited States; 3 Department of Internal Medicine, University of MichiganAnn ArborUnited States; Yale-NUS CollegeSingapore; Weill Cornell MedicineUnited States

**Keywords:** aging, healthspan, *D. melanogaster*, *C. elegans*, insulin signaling, sensory perception

## Abstract

As the demographics of the modern world skew older, understanding and mitigating the effects of aging is increasingly important within biomedical research. Recent studies in model organisms demonstrate that the aging process is frequently modified by an organism’s ability to perceive and respond to changes in its environment. Many well-studied pathways that influence aging involve sensory cells, frequently neurons, that signal to peripheral tissues and promote survival during the presence of stress. Importantly, this activation of stress response pathways is often sufficient to improve health and longevity even in the absence of stress. Here, we review the current landscape of research highlighting the importance of cell non-autonomous signaling in modulating aging from *C. elegans* to mammals. We also discuss emerging concepts including retrograde signaling, approaches to mapping these networks, and development of potential therapeutics.

## Introduction

It is estimated that by 2050 the number of US citizens over the age of 65 will reach nearly 100 million, more than twice as many as today (United Nations, 2015). If this increase occurs without significant fiscal and structural changes, the cost of this aged population could cripple economies across the world. Therefore, deciphering and mitigating the aging process to create a healthier older population has become an increasingly important goal within biomedical research. The benefits of discovering therapeutics that target aging are many, including (1) decreasing the financial burden on our strained healthcare system, (2) increasing the amount of time older adults live free of chronic diseases (often denoted as healthspan), and (3) potentially increasing maximum human lifespan.

Organismal lifespan was first presented as a genetically modifiable trait by groundbreaking publications from the Johnson, Kenyon and Ruvkun labs describing the effects of the FOXO transcription factor DAF-16 on longevity in *Caenorhabditis elegans* (Friedman and Johnson, 1988; Kenyon et al., 1993; Kimura, 1997). These findings played a critical role in the field’s current interest in identifying signals that are crucial regulators of aging across the entire organism. Additional studies have shown that environmental factors, such as food perception or oxygen levels (Libert et al., 2007; Kaeberlein et al., 2006; Mehta et al., 2009), can also modify longevity in model organisms. Although modifying genes or substantially changing environments is not plausible in humans, it is feasible to find anti-aging therapeutics that mimic environmental cues or genetic signaling environments.

Deciphering how cells relay information to one another remains one of the foundational discoveries in biology. It was first posited by John Langley that cells express receptor proteins on the extracellular side of the cell membrane and, when bound by a signaling molecule, initiate a downstream physiological response (Maehle, 2004). This was validated by a series of discoveries, starting with Rita Levi-Montalcini’s finding of nerve growth factor in the 1950s and continuing with the discovery of other growth factors (Cohen, 1965) before eventually finding the receptors themselves (Paton and Rang, 1965). These discoveries were pivotal in furthering our understanding of cellular patterning during development (Nusse, 2003) as well as how organisms adapt to external stimuli. This concept, that cells can relay critical information to other cells in response to an initial signaling cue, allows for genes expressed in one cell or tissue to affect the physiology of other cells and tissues. This ability of genes to affect processes outside of the cells they are expressed in is often referred to as cell non-autonomous action or signaling.

More recently, high-profile publications from multiple labs have shown that many signaling pathways reported to improve longevity (e.g. mitochondrial stress, insulin-like signaling, heat shock, and the hypoxic response) act through cell non-autonomous signaling mechanisms (Schinzel and Dillin, 2015; Leiser et al., 2015; Zhang et al., 2019; Zhang et al., 2013; Riera et al., 2014; Ro et al., 2016). These pathways originate in sensory cells, often neurons, that signal to peripheral tissues and promote survival during the presence of stress. Importantly, this activation of stress response pathways, either through genetic modification or exposure to environmental stress, is often sufficient to improve health and longevity. Additionally, genetic modification of these pathways can often target the aging process while sparing growth/development/reproduction effects that are often consequences of environmental stress. Understanding how cell non-autonomous signaling influences longevity is a relatively recent concept in aging research and presents a novel opportunity to discover pharmacological interventions that modulate signaling to increase healthspan and longevity.

In this review, we summarize the recent wave of studies investigating the effects of cell non-autonomous signaling on a myriad of canonical aging pathways across taxa. Further, we discuss where the field has excelled and what we can learn from other areas of research that have successfully mapped the neuronal circuitry of behavioral phenotypes.

## Caenorhabditis elegans

Utilization of the model organism *C. elegans* has played an integral role in bringing the biology of aging field into prominence. The discovery that mutations in the sole nematode insulin-like growth factor receptor (IGFR), *daf-2*, can double a multicellular organism’s lifespan, launched a new field (Friedman and Johnson, 1988; Kenyon et al., 1993; Kimura, 1997). Their discrete, well-defined somatic cell fate makes them an ideal model system to study how cell non-autonomous signaling influences a complex phenotype like aging. We begin by discussing the extensive studies of different *C. elegans* pathways that protect against stress and modify aging through cell non-autonomous mechanisms.

### Energy balance and insulin signaling

**Figure 1.**
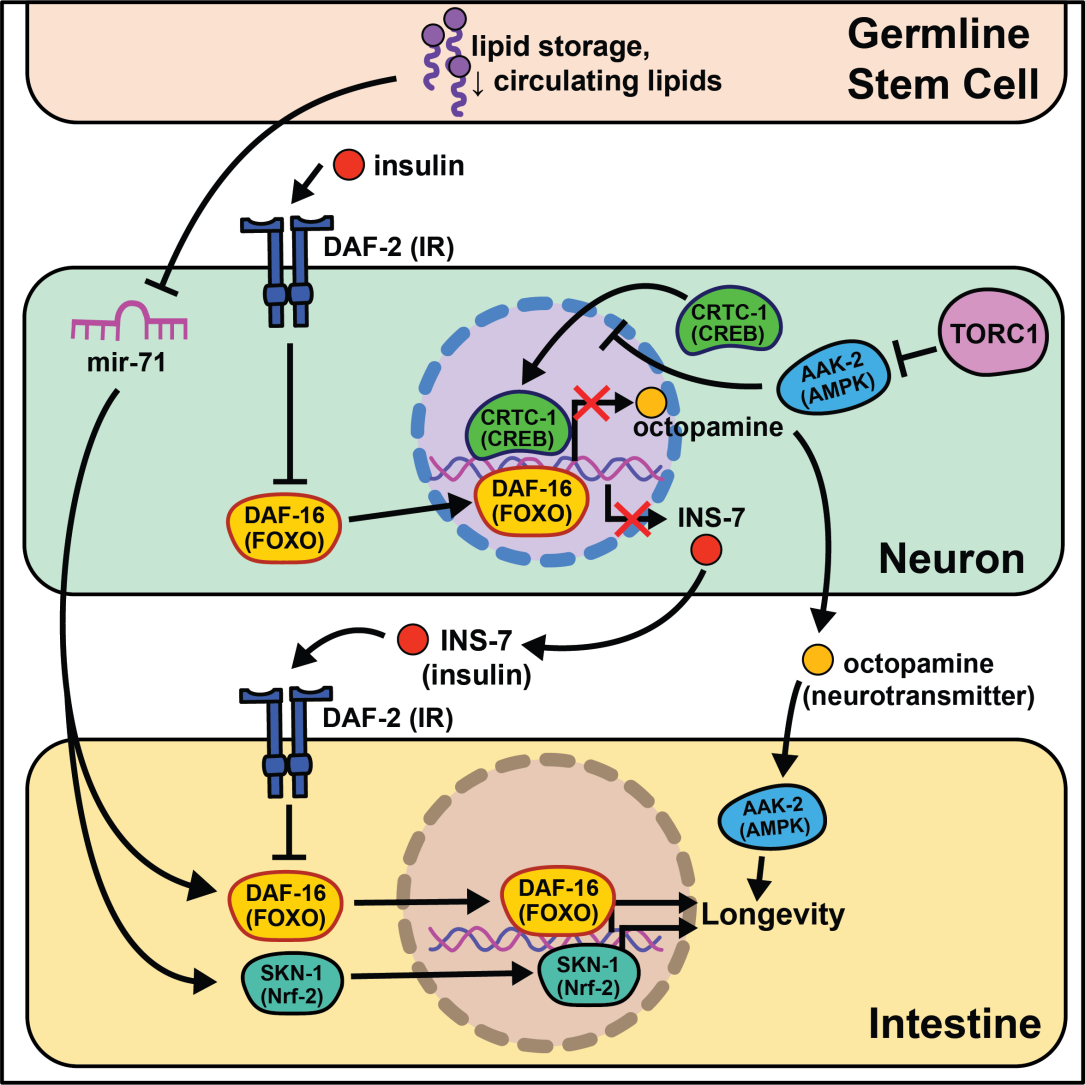
Summary of the role energy balance and insulin signaling on cell non-autonomous modulation of longevity in *C. elegans*. Mammalian orthologs are listed in parentheses. If there are no parentheses, the name is shared across taxa.

Soon after the initial discovery that diminished insulin signaling can extend lifespan, the aging field began exploring where decreased insulin signaling was required to promote longevity. By constructing animals with mosaic expression of *daf-2*, several cell lineages were identified that require *daf-2* mutations to reproduce the entirety of the *daf-2* lifespan benefit (Apfeld and Kenyon, 1998). This study was the first to clearly define a role for cell non-autonomous activity in aging and validate the significance of inter-tissue signaling during the lifespan of an organism. From here they sought to understand the effects of insulin signaling across tissues and the emergent role of the nervous system in influencing longevity came to the forefront of the field (Figure 1).

The identification of this role began through epistasis experiments. It was discovered that *daf-2* mutants completely require the class O of forkhead box transcription factors (FOXO) ortholog *daf-16* to extend lifespan (Gottlieb and Ruvkun, 1994), suggesting that *daf-16* nuclear localization and transcriptional activity is responsible for the longevity benefits. While most of *daf-16*’s pro-longevity effects are thought to be tied to cell-autonomous transcription of target genes in the intestine (Libina et al., 2003), more recent studies show that a subset of target genes, *dod-8, dod-11,* and *hsp12.6*, influence muscle aging cell non-autonomously (McCormick et al., 2012). Intestinal expression of *daf-16* leads to elevated induction of target genes in the nervous system and hypodermis. Conversely to *daf-16,* tissue-specific expression of *daf-2* or *phosphatidylinositol 3-kinase homolog age-1* in neurons is sufficient to partially block lifespan extension in mutant animals, whereas *daf-2* or *age-1* rescue in muscle is sufficient to restore metabolic function (Wolkow et al., 2000). These studies provide evidence that metabolism and aging are separable, yet interrelated, and that bi-directional signaling from metabolically active tissues to the nervous system occurs. We will expand on this understudied phenomenon in the Emerging concepts section.

Nematodes have amphid neurons with ciliated projections that relay information about their current environment to the worm. Interestingly, when these projections are genetically knocked out or laser ablated, the animals live longer, often with the requirement of *daf-16* activity (Apfeld and Kenyon, 1999; Alcedo and Kenyon, 2004). These data are supported by a recent study analyzing RNA in isolated neurons of *daf-16* and *daf-16; daf-2* mutants that finds *daf-16* target genes exclusively expressed in mechanosensory neurons (Kaletsky et al., 2016). This led to identification of a novel gene, *fkh-9/*FOXL1, that, when knocked out, entirely abrogates *daf-2* lifespan. Another transcriptional microarray analysis identified a set of neuronally expressed genes upregulated in animals on *daf-2* RNAi and downregulated in animals on *daf-16* RNAi (class I) or vice versa (class II) (Silva et al., 2013). The class II hit, *ins-7*, predicted to encode for an insulin-like peptide, is required for *daf-2* RNAi-mediated lifespan extension. *ins-7* may act as a signaling molecule in insulin-mediated cell non-autonomous signaling from the nervous system to the intestine. These data are compelling as *C. elegans* are predicted to have 40 insulin-like peptides that both agonize and antagonize *daf-2,* some of which modify the lifespan of wild-type animals when neuronally overexpressed (De Rosa et al., 2019).

Foundational work from the Kenyon lab established that signaling from germ cells regulates insulin signaling by decreasing *daf-16* activity throughout the organism (Hsin and Kenyon, 1999). This means that ablating the germline genetically or physically leads to *daf-16* activation and increased longevity. The absence of germline signals activates a *daf-12*-dependent sterol signaling pathway in somatic reproductive tissues (Yamawaki et al., 2010). This pathway induces the expression of *lips-17/fard-1* and produces an unknown lipophilic signal to increase *sod-3/dod-8* in other somatic tissues (McCormick et al., 2012). This signaling pathway is an illustration of cells/tissues signaling to other tissues that ‘times are good/bad’.

Despite the groundbreaking discovery of microRNAs (miRNAs) in *C. elegans* (Reinhart et al., 2000), their size, abundance across the genome, and evolutionary conservation, little work has investigated their potential role in cell non-autonomous modulation of aging. An intriguing study begins this exploration, interrogating how *mir-71* modulates lifespan (Boulias and Horvitz, 2012). *mir-71* is necessary for lifespan extension in *glp-1* germline mutants, while *mir-71* overexpression in germline mutants extends lifespan beyond that of germline mutations alone. Rescuing *mir-71* in neurons alone is sufficient to rescue the germline mutant longevity phenotype, and intestinal *daf-16* expression is necessary for *mir-71* overexpression to extend lifespan in germline mutants. While this study presents an incomplete picture, it provides sound evidence for future studies to consider miRNAs as a target for inter-tissue signaling during the aging process.

The absence of gonadal stem cell signaling leads to activation of intestinal transcription factors like *skn-1/*Nrf2 that help to globally remodel the organism’s metabolism (Steinbaugh et al., 2015). A lack of gonadal stem cells also increases circulating fatty acids (FAs) that are normally deposited in this tissue. This increase in circulating lipids may induce *skn-1/*Nrf2 nuclear localization that, in turn, enhances lipid metabolism and increases lifespan. *nhr-49*/HNF4α, a nuclear hormone receptor widely expressed throughout somatic tissues, also regulates both *glp-1-*mediated longevity and lipid metabolism (Ratnappan et al., 2014). Furthermore, *nhr-49* overexpression extends lifespan in a *daf-16*- and *glp-1*-dependent manner. While this work cannot be directly translated to human health, these data provide initial evidence for critical signaling events between stem cells and nearby somatic tissues that influence longevity.

Multiple nutrient-sensing pathways that interact with insulin signaling are conserved from worms to mammals including the target of rapamycin (TOR) and AMP-activated protein kinase (AMPK) pathways. The latter nematode ortholog, *aak-2*, has increased activity under low energy conditions and is sufficient to promote longevity when overexpressed (Apfeld et al., 2004). Follow-up studies show that *aak-2* activity suppresses cAMP-response element binding protein (CREB)/CREB-regulated transcription coactivator 1 (CRTC-1) transcriptional regulation and this ultimately extends lifespan (Mair et al., 2011). *crtc-1* neuron-specific knockout extends lifespan without the undesirable pleiotropic effects that accompany AMPK activation, like decreased growth and fecundity (Burkewitz et al., 2015). Interestingly, *nhr-49/*HNF4α is activated by AAK-2 (Moreno-Arriola et al., 2016), and its neuronal expression is required for the lifespan extension in AAK-2 overexpressing animals. Furthermore, neuronal CRTC-1 is sufficient to block AMPK-mediated longevity. Neuronal *crtc-1* levels modify intestinal AAK-2 activity through the neurotransmitter octopamine as a mechanism for the nervous system to convey ‘times are good’. Despite these results, many questions remain involving this signaling pathway. Octopamine is thought to be exclusively synthesized in the RIC head neurons (Alkema et al., 2005) and the two known octopamine receptors, *ser-3* and *octr-1,* are expressed throughout inner neurons but not in peripheral tissues (Suo et al., 2006; Mills et al., 2012). This suggests that octopamine acts as an initiating signal instead of a direct signal from neurons to downstream tissues. Recent work highlights this point by showing GABAergic signaling and neuron excitability inversely changes throughout the life of short- and long-lived animals (Wu et al., 2018).

TOR and AMPK play antagonistic roles in modulating lifespan and metabolism. Downregulation of TORC1 complex proteins, like *raga-1*, increases lifespan (Schreiber et al., 2010) but requires neuronal *aak-2* activity (Zhang et al., 2019). Moreover, neuronal TORC1 expression is sufficient to shorten lifespan, consistent with a central ‘times are good’ signal modulating longevity. RNA-seq comparisons of wild-type to *raga-1* knockout and neuronal overexpression identified *unc-64/*syntaxin as epistatic to neuronal *raga-1* activity. This work supports neural signaling as responsible for metabolic rearrangements, like increased mitochondrial fragmentation, that occur in peripheral tissues.

Although many of these studies identify the existence of neurosignaling pathways that drive metabolic changes in peripheral tissues, few have identified the signal(s) through which the nervous system transmits information to these tissues. Probing the precise mechanisms of nervous system to intestinal signaling will be an essential next step in furthering our understanding of how cell non-autonomous signaling influences aging.

### Proteostasis signaling pathways

A significant body of literature links increased stress resistance with longevity, leading to the hypothesis that acute moderate stress can trigger hormetic effects that extend lifespan (Calabrese et al., 2015). Eukaryotic cells have evolved several organelle-specific stress responses that, when induced, extend nematode lifespan.

Knock-down of complex IV of the electron transport chain (ETC) with *cco-1* RNAi leads to delayed growth, slowed movement, reduced body length, and increased lifespan. Importantly, *cco-1* knock-down exclusively in neurons increases lifespan without pleiotropic phenotypes by altering mitochondrial homeostasis in peripheral tissues (Durieux et al., 2011). Interestingly, this lifespan extension requires the mitochondrial unfolded protein response (mt-UPR), but not *daf-16*/insulin signaling. A follow-up study using the heat-shock protein-6 (*hsp-6)*p::GFP transcriptional reporter identified *vps-35* as lacking peripheral mt-UPR (Zhang et al., 2018a). VPS-35 is a highly conserved protein in the retromer complex, involved in recycling Wnt and the Wnt secretion factor MG-4 (Prasad and Clark, 2006). In agreement with this, the Wnt receptor *egl-20* is necessary and sufficient for cell non-autonomous mt-UPR induction and longevity. Interestingly, neuronal serotonin production is necessary for cell non-autonomous mt-UPR even though the loss of each of the four known serotonin receptors (*ser-1, ser-4, ser-7*, and *mod-1*) has no effect. These results clearly define that mitochondrial stress, through reduction of ETC activity or separate activation of the mt-UPR, can be transmitted by neurons cell non-autonomously to modify aging. As with many of these neuronal-based networks, however, the central signaling pathways remain largely uncharacterized.

Multiple labs find that neuronal activation of the endoplasmic reticulum unfolded protein response (ER-UPR) is sufficient to enhance stress resistance and extend lifespan (Taylor and Dillin, 2013; Imanikia et al., 2019b; Frakes et al., 2020; Daniele et al., 2020; Imanikia et al., 2019a). This crucial discovery stems from identifying that the constitutively active spliceoform of X-box binding protein 1 (*xbp-1s)*, a transcription factor activated by the ER-UPR, rescues older animal survival on paraquat (Taylor and Dillin, 2013). Expression of *xbp-1s* in the nervous system or intestine extends lifespan, and exclusive *xbp-1s* expression in the neurons is sufficient to increase paraquat stress resistance in young and older animals. It is likely that neuronal ER-UPR releases an activation signal conferring *xbp-1s* upregulation in the intestines that rescues motility in models of proteotoxicity like Aβ, polyglutamine aggregates (Q40), and dynamin (Imanikia et al., 2019b).

More recent work begins to parse out where *xbp-1s* is required in the nervous system to extend lifespan. Surprisingly, expression of *xbp-1s* in glia, helper, and insulator cells for neurons, extends lifespan and activates the ER-UPR in peripheral tissues (Frakes et al., 2020). This *xbp-1s*-mediated signal requires neuropeptide signaling but not neurotransmission for intestinal induction of *hsp-4,* suggesting a neuropeptide is the intermediate signal from the glia cells to the intestine (Frakes et al., 2020). While it is useful to rule-out neurotransmitters as the causative signaling molecules, there remain hundreds of potential coding regions annotated as ‘neuropeptides’ in *C. elegans* (Li et al., 1999a; Li et al., 1999b).

**Figure 2.**
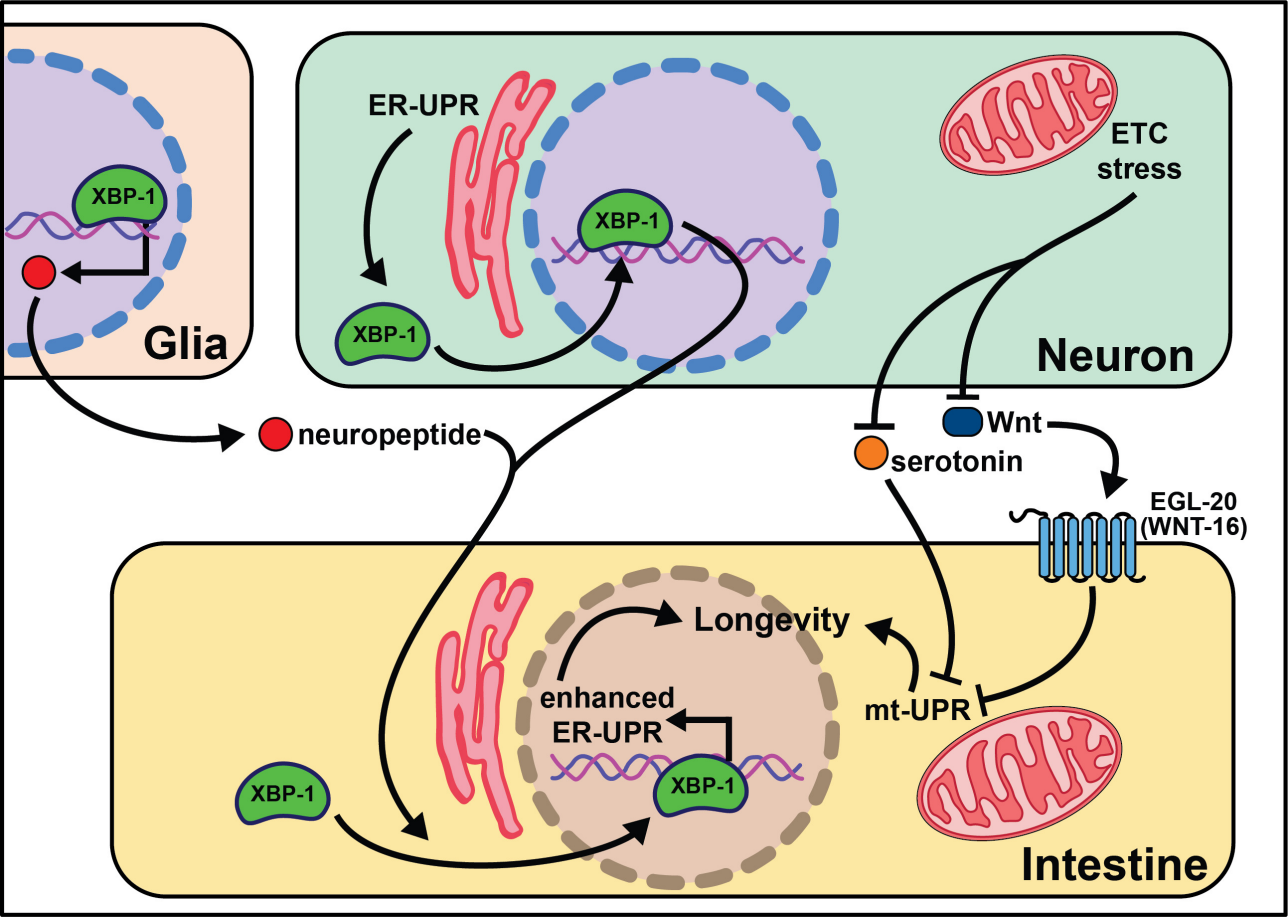
Summary of the role of proteostasis in cell non-autonomous modulation of longevity in *C. elegans*. Mammalian orthologs are listed in parentheses. If there are no parentheses, the name is shared across taxa.

Another open question surrounds whether other transcription factors that respond to the ER-UPR can recapitulate the effects of *xbp-1* activation. Preliminary findings point to *xbp-1* activity, not *pek-1* or *atf-6,* two transcription factors also activated by the ER-UPR (Frakes et al., 2020), as key to modifying lifespan. This implies that uncovering the pro-longevity targets of XBP-1 will be of high interest to understand and translate these results. Neuronal activation of *xbp-1s* results in changes in fat metabolism, a build-up in oleic acid (OA), and leaner animals (Imanikia et al., 2019a). It is possible that the pro-longevity phenotypes associated with *xbp-1s* are due to increased expression of lysosomal genes, like *asp-13* and *vha-18*, that enhance acidity and, therefore, protease activity. Furthermore, the lifespan phenotype and expansion of the ER in *xbp-1s* animals requires lipid depletion (Daniele et al., 2020). Initial results show enhanced intestinal lipid depletion through *ehbp-1* overexpression modestly extends lifespan and partially recapitulates the effects of *xbp-1s*. Taken together, these data emphasize the significant role metabolically active tissues, like the intestine, play in properly responding to cues from the nervous system (summarized in Figure 2). While the ER-UPR field is beginning to narrow in on specific neuronal cells and signaling molecules, much is left to delineate in the neuronal circuitry. Understanding how, when, and where neuromodulators affect normal and pro-longevity conditions is paramount in translating these results to higher organisms and discovering therapeutic treatments to mimic these phenomena.

### Perception of external stimuli

**Figure 3.**
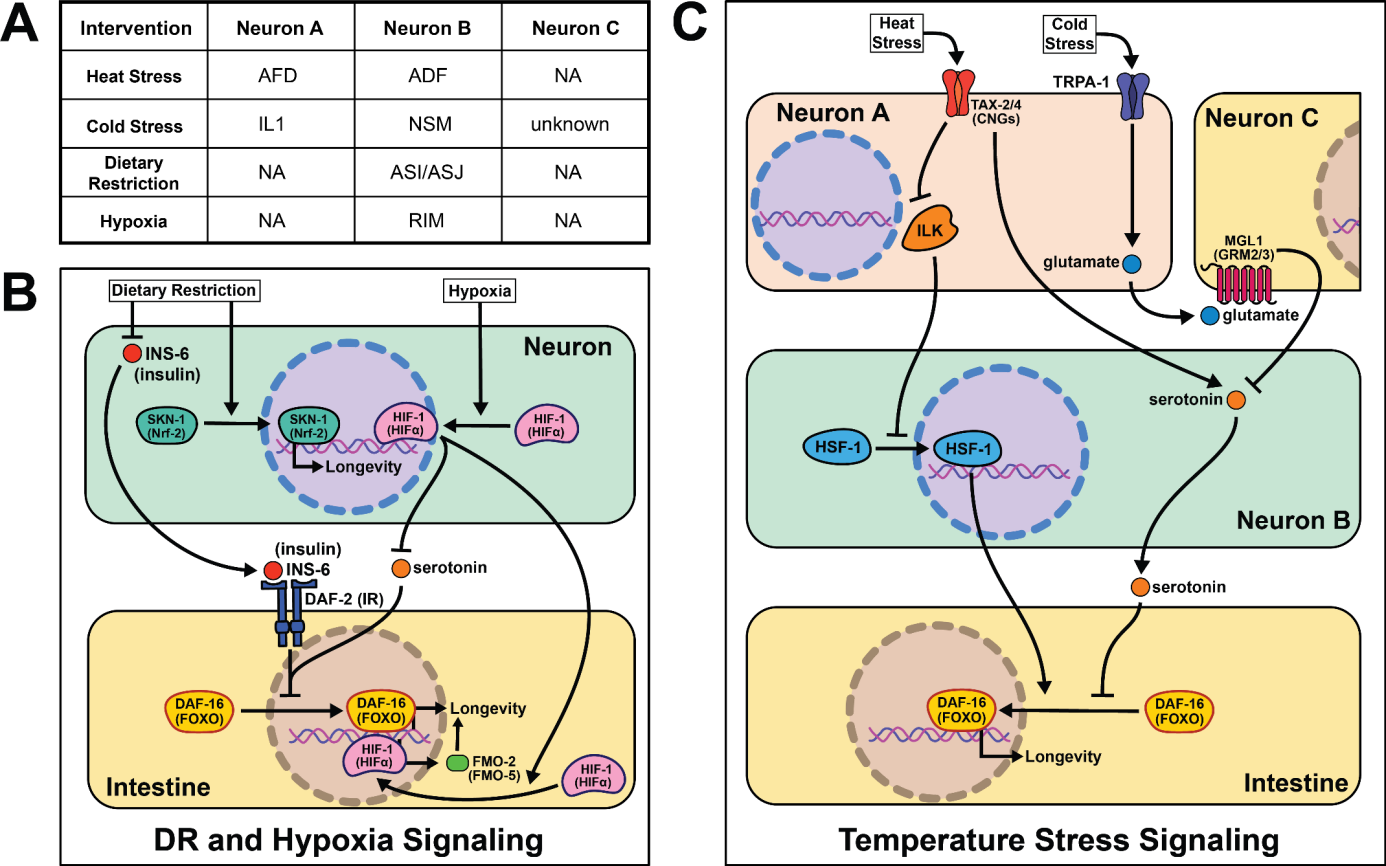
Summary of the role of perception on cell non-autonomous modulation of longevity in *C. elegans.* Mammalian orthologs are listed in parentheses. If there are no parentheses, the name is shared across taxa.

An organism's ability to respond to changes in the environment, such as temperature, oxygen levels, and smells, is vital to their survival. In this section, we chronicle research findings linking perception and organismal aging (Figure 3).

As poikilotherms, *C. elegans* are responsive to heat- and cold-shock. Generally, longevity varies inversely with temperature, where animals housed at lower temperatures (15℃) live longer than those at higher temperatures (25℃). Based on our understanding of thermodynamics, this observation seems intuitive, but several papers have challenged this theory and shown changes in lifespan across temperature are genetically modulated. In fact, many pro-longevity genetic mutations have a distinct relationship with temperature, promoting longevity at either cold or warm temperatures but rarely both (Miller et al., 2017). These data are consistent with many genes playing an active role in the physiological response to changes in temperature. Whether by modifying the perception or response to temperature, this interplay suggests that genes actively influence lifespan at various temperatures, refuting thermodynamics as the sole influence on temperature-mediated lifespan changes.

A robust body of research surrounds activating the heat-shock response (HSR) and longevity. In *C. elegans*, post-reproductive adulthood is accompanied by an abrupt decline in protein quality control (Walther et al., 2015; Labbadia and Morimoto, 2015; Santra et al., 2019). Thus, maintaining the ability to effectively respond to heat stress remains a hallmark of long-lived animals. It is important to note that heat shock, described in the literature as >30℃, likely uses partially distinct genetic mechanisms from warmer conditions (22℃−25℃) that will be addressed separately.

Early studies implicate the nervous system in regulating proteostasis through the HSR. Key signaling components of amphid neurons, *gcy-3* and *ttx-3*, are necessary for proper induction of global HSR during heat shock (Prahlad et al., 2008). These early studies also implicate an unknown neurotransmitter in signaling from the nervous system to promote survival during heat shock. Optogenetic stimulation of the AFD thermosensory neurons triggers serotonin release from the ADF neurons that activates heat shock factor 1 (HSF-1) in the germline in the absence of heat shock (Tatum et al., 2015). In the absence of *ser-1*, a 5-HT2B serotonin receptor ortholog, HSF-1 is not re-localized to the nucleus upon AFD optogenetic excitation or heat shock. This result suggests that *ser-1* is necessary for proper signaling during HSR. It is worth noting there is no predicted synapse or gap junction between AFD and ADF neurons, meaning there is likely an intermediate signaling molecule(s) and/or cells yet to be discovered.

While HSR proteins are necessary for animals to survive warmer temperatures (Lee and Kenyon, 2009), it is significant that overexpression of *hsf-1* in neurons is sufficient to increase stress resistance and longevity (Douglas et al., 2015). Linking back to earlier work, intact signaling from the AFD/AIY neurons is required for heat stress resistance in worms overexpressing *hsf-1* (Prahlad et al., 2008). This may be through *pat-4/* integrin linked kinase (ILK) activity in the AFD/AIY (Kumsta et al., 2014). Interestingly, intestinal *daf-16* activity is required for the lifespan phenotype but unnecessary for heat stress resistance (Douglas et al., 2015), suggesting these phenotypes have distinct signaling events despite their positive correlation.

Less is known about the mechanisms of cold- and warm-sensory signaling and metabolic remodeling that promotes longevity. As with the HSR, the AFD neurons are also thought to play a role in appropriately responding to warmer temperatures as laser ablation and genetic disruption exclusively shortens lifespan at 25°C (Lee and Kenyon, 2009). Mutants lacking functional CNG calcium channels, a *tax-2/tax-4* heterodimer, are also short-lived at 25°C and thought to be necessary for AFD neuronal activity when exposed to warm temperatures. These results are corroborated in a recent study showing the ASJ sensory neurons also require functional *tax-2/tax-4* channels to sense warm temperatures and activate intestinal *daf-16* to extend lifespan (Zhang et al., 2018a; Artan et al., 2016). Experimental evidence suggests once these thermosensory neurons are activated they deploy *daf-9*, a cytochrome P450 ortholog, which inhibits *daf-12*, a nuclear hormone receptor, allowing worms to live longer at 25°C (Lee and Kenyon, 2009). An important distinction remains between AFD activation during warm- and heat-shock as many heat shock proteins, like *hsp-60* and *hsp-70*, are not upregulated at 25°C (Lee and Kenyon, 2009). These data suggest that perception and response to temperature through thermosensory neurons are sufficient to modulate aging across temperatures. They also refute thermodynamics as the sole mechanism for how poikilotherms live shorter at higher temperatures.

In agreement with neural modulation of aging in warmer temperatures, a transient receptor potential (TRP) channel, TRPA-1, detects cold temperatures in chemosensory neurons. TRPA-1 signals through a protein kinase C (PKC) ortholog, PKC-2, to increase intestinal DAF-16 activity and therefore lifespan (Xiao et al., 2013). Loss of *trpa-1* channels in the nervous system prevents the lifespan increase observed in wild-type worms at cooler temperatures (15–20°C), but does not change longevity at warmer temperatures (25°C). Interestingly, human transgenic TRPA-1 recapitulates many of these findings, suggesting a conservation in function. An unbiased screen of all sensory neurons showed *trpa-1* expression in the head neuron IL1 is necessary and sufficient to rescue *trpa-1* knockout. Further, genetically knocking out glutamate secretion and uptake prevents IL1 from modifying lifespan (Zhang et al., 2018b). MGL-1, the glutamate receptor implicated in this study, is only expressed in neurons, indicating there must be another neuron involved in this pathway. Knocking out serotonin signaling blocks the effects of IL1 on lifespan, and transgenic serotonin expression in the NSM enteric neuron rescues the phenotype. The intestinal GPCR *ser-7* is the likely downstream receptor responding to serotonin release. This study offers a more complete model than is often presented in the field, and suggests it is feasible for other cell non-autonomous signaling pathways to be more explicitly characterized in future studies.

The nervous system in *C. elegans* plays a crucial role in determining nutrient quality and safety as they forage for food, and interestingly, the lack of any perceived signal (i.e. dietary restriction (DR)), can also act as a signal on its own. DR, first reported to increase lifespan in rats in 1935 (McCay et al., 1935), is an intervention that significantly limits food intake without malnutrition, and has been the most consistent intervention to increase longevity across species (Fontana and Partridge, 2015). However, since implementation of any dietary intervention for humans at a whole population level is challenging, mapping out the molecular and signaling mechanisms downstream of food perception, where they can be targeted directly, circumvents the challenges of adopting population level-DR protocols.

The first report of DR acting through a sensory, cell non-autonomous signaling mechanism in worms was in 2007. They identify the antioxidant response transcription factor skinhead 1 (SKN-1) in ASI sensory neurons as leading to increased whole-body respiration and extended lifespan (Bishop and Guarente, 2007). This report remains foundational in establishing food perception, or lack thereof, as a major driver of the health benefits of DR. More recent studies corroborate the significance of sensory neurons when nematodes are subjected to long- and short-term starvation. A subset of sensory neurons, the ASI and ASJ, shorten lifespan through the expression of insulin-like peptide (ILP) *ins-6* during food perception (Artan et al., 2016). More specifically, *ins-6* is released upon feeding, and *ins-6* overexpression exclusively in the ASI or ASJ neurons blocks the longevity effects of *tax-2; tax-4* mutant worms when fasted. This suggests that an unknown inverse signal may suppress *ins-6* expression during fasting.

Sensory cues from food perceived by the nervous system trigger a host of behavioral and metabolic rearrangements that accompany changes in lifespan. Particularly, in the absence of food, nematodes tend to increase their movement when foraging and stop pharyngeal pumping (You et al., 2008; McCloskey et al., 2017; Waggoner et al., 1998). Once an attractive odorant is perceived, serotonin is released triggering triglyceride fat catabolism by a predicted acyl-CoA oxidase, *acox-1* (Srinivasan et al., 2008). Surprisingly, this fat catabolism is necessary and sufficient to modulate changes in behavior. *acox-1* mutants do not respond with the canonical behavioral changes observed when animals are exposed to exogenous serotonin (Zheng et al., 2018). Additionally, rescuing *acox-1* expression in the intestine alone abrogates the fat accumulation and pumping response to serotonin exposure. *egl-2*, an ether-à-go-go (EAG) K+ channel expressed in the sensory neurons, likely functions as an intermediary neuron signaling back to serotonin neurons communicating satiety from peripheral tissues. Intriguingly, antagonizing serotonin signaling through an atypical antidepressant mianserin extends lifespan and is non-additive with DR (Petrascheck et al., 2009). It is worth exploring whether the changes in metabolism and lifespan when serotonin signaling is antagonized are acting within the same pathway. These results corroborate the hypothesis that a feed-forward signal is released from the intestine back to the nervous system during food perception (see section Emerging concepts).

Recognition of low-oxygen conditions or genetic stabilization of the conserved hypoxia-inducible factor-1 (HIF-1) extends nematode longevity (Mehta et al., 2009; Leiser et al., 2013). A single protein, flavin-containing monooxygenase-2 (FMO-2), is necessary and sufficient to provide many of the benefits of HIF-1 activation (Leiser et al., 2015). Neuronal stabilization of HIF-1 is sufficient to induce intestinal *fmo-2* and improve health and longevity. Serotonergic signaling is required for HIF-1-mediated longevity and *fmo-2* induction, while FMO-2 overexpression in the intestine is sufficient to increase lifespan. Interestingly, other researchers have found FMO induction in multiple mammalian models with increased lifespan, including DR, consistent with FMOs playing a conserved role in promoting long-term health and increasing the likelihood these results will be translatable to human longevity (Swindell, 2009; Steinbaugh et al., 2012).

Together, perception of many environmental signals influence lifespan in *C. elegans*. While the mode of sensory detection varies between these environmental signals, perception of temperature, oxygen, food availability, and mechanosensory cues all activate sensory neuron-initiated signaling pathways. This external perception interacts with internal cues (e.g. germline signal, proteostasis, and metabolism) to appropriately respond and modify physiology. While we have begun to identify the cells and signals involved in these sensory-driven longevity pathways, many questions remain regarding how information is transmitted and interpreted both within and between tissues.

## Drosophila melanogaster

While *C. elegans,* with its simplicity and finite number of cells, is perhaps the most powerful system for identifying genetic mechanisms of aging, it does pose some limitations: its nervous system is rudimentary, precise manipulation of diet and other environmental factors is difficult, and its small behavioral repertoire is restrictive. Conversely, studies of cell non-autonomous modulation of aging in vertebrate animals are impeded by multiple factors, including but not limited to: (1) the time required for measuring lifespan, (2) challenges in using large-scale genetic modification for pathway discovery, and (3) the difficulty of identifying small subsets of key cells within substantially larger and more complex tissues. Given the remarkable advances in neuroscience, together with its long-standing success as a model for both behavioral neuroscience and aging biology, the vinegar fly, *Drosophila melanogaster*, provides unique strengths to investigate these questions. Conservation of mechanisms of aging, including insulin/FOXO-related signaling and sensory-derived control of longevity, in worms, flies, and mammals suggests that signaling mechanisms likely become more complex in higher organisms but produce similar pro-longevity outcomes. Although the phylogenetic relationship between nematodes, arthropods, and vertebrates is debated, 18S rRNA and mitochondrial rDNA sequencing suggests a greater evolutionary distance between *D. melanogaster* and *C. elegans* than between *D. melanogaster* and *M. musculus* (Fitch, 2005). Consequently, mechanisms of aging conserved between worms and flies are also likely to span the smaller evolutionary gap between flies and mammals. In the following sections, we provide an overview of key cell non-autonomous modulators of fly health and aging (Hwangbo et al., 2004; Libert et al., 2007).

### Energy balance and insulin signaling

**Figure 4.**
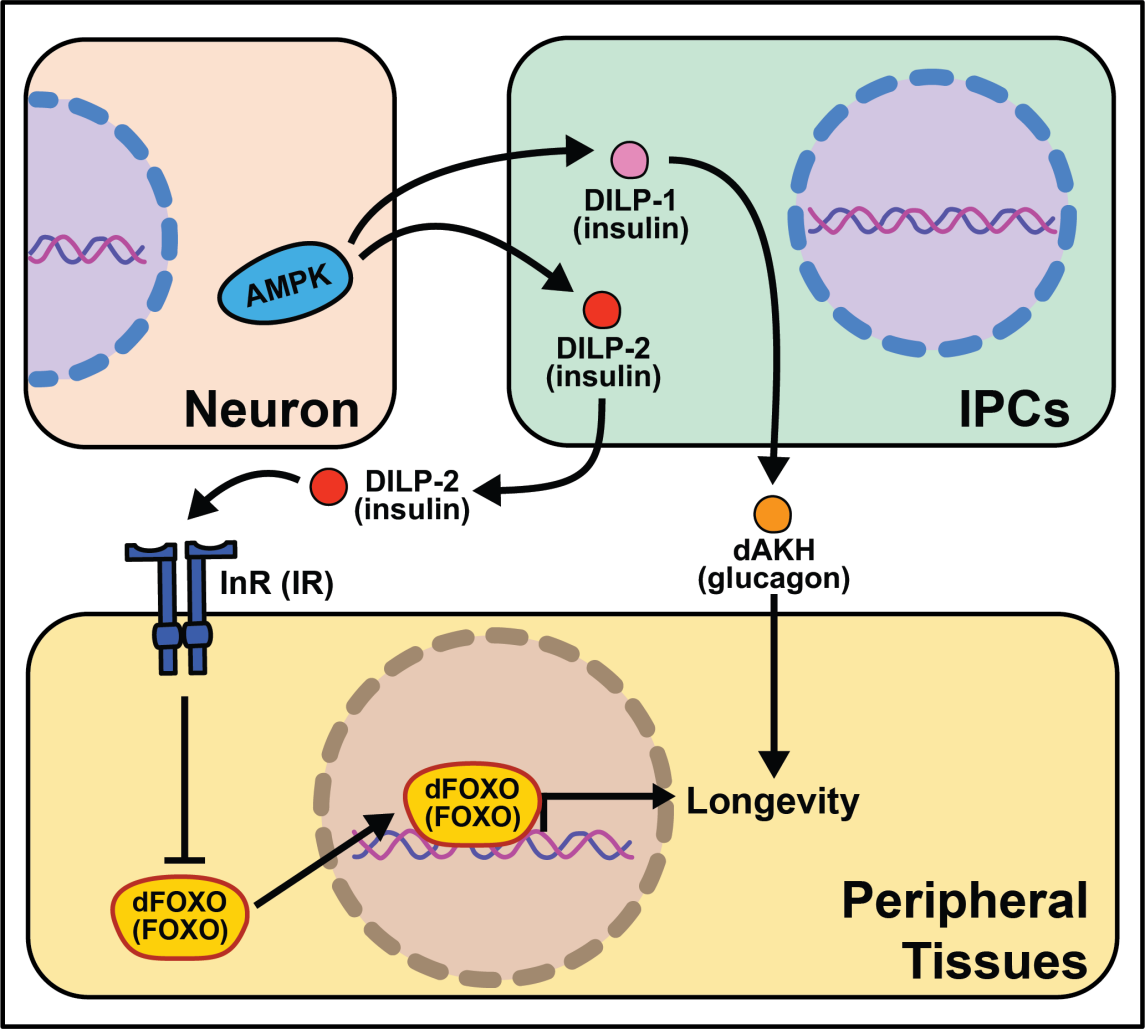
Summary of the role energy balance and insulin signaling on cell non-autonomous modulation of longevity in *Drosophila melanogaster*. Mammalian orthologs are listed in parentheses. If there are no parentheses, the name is shared across taxa.

Soon after the foundational discovery that reduced insulin signaling increases nematode lifespan, experiments in *Drosophila* revealed that such results were not worm-specific and that this pathway may be involved in modulating aging across taxa (Kannan and Fridell, 2013). Mutations disrupting molecules in this pathway such as the single insulin receptor, *dInR*, or the fly homolog of the insulin receptor substrate, *chico*, exert non-autonomous effects on aging, where reduced insulin/IGF-1 signaling (IIS) is associated with extended lifespan (Clancy et al., 2001; Tatar et al., 2001). Genetic manipulations that mimic reduced IIS, such as overexpression of *dFOXO* in the abdominal or pericerebral fat body or overexpression of phosphatase, *dPTEN*, in the pericerebral fat body also extend fly lifespan (Giannakou et al., 2004; Hwangbo et al., 2004). These data indicate that insulin signaling acts cell non-autonomously to control aging and promotes bi-directional signaling between peripheral tissues and neurons like what is seen in *C. elegans* (Figure 4). 

The *Drosophila melanogaster* genome encodes several insulin‐like peptide genes (*dilps*), which are produced in a handful of neurosecretory cells in the *pars intercerebralis* region of the fly brain. These insulin producing cells, called IPCs, release the signaling molecules into the hemolymph to signal to the rest of the body through a single insulin‐like receptor (InR) (Brogiolo et al., 2001; Colombani et al., 2012; Grönke et al., 2010). Among their range of biological functions in development and physiology, *dilps* modulate aging, and in this context, the most well studied are *dilp2*, *dilp3*, *dilp5,* and *dilp6* (Bai et al., 2012; Broughton et al., 2010; Grönke et al., 2010). Partial ablation of IPCs, the production site of DILPs 2, 3, and 5 reduces IIS and increases lifespan (Broughton et al., 2005; Haselton et al., 2010). Knocking-down GABA-B receptors in IPCs decreases DILP secretion in fed and fasted conditions and yields a small but significant decrease in stress resistance and lifespan under starvation (Enell et al., 2010). Mutation of *dilp2*, *dilp3,* and *dilp5 together increases lifespan as does loss of dilp2* alone, although loss of other individual *dilps* does not (Grönke et al., 2010). Induction of *dilp6* in fat body tissue promotes longevity. It is unclear whether this is a direct effect of DILP6 or is due to a decrease in the secretion of DILP2 from the IPCs (Bai et al., 2012). Similarly, the extended lifespan observed following increased *dFOXO* expression in pericerebral fat body may also result from a decrease in *dilp2* expression in the IPCs (Wang et al., 2005). More recent work has revealed a role for *dilp1* in promoting lifespan (Post et al., 2019), potentially through induction of adipokinetic hormone (AKH), which is a functional homolog of mammalian glucagon (Bednarova et al., 2013) and which increases fly lifespan, fat metabolism, and free fatty acid catabolism (Waterson et al., 2014; Waterson et al., 2015).

The link between lifespan extension from manipulation of neuronal dilps and the insulin-responsive transcription factor FOXO in peripheral tissues are less clear in *Drosophila* as in *C. elegans*, suggesting that IIS extends lifespan, at least partly, through FOXO‐independent pathways. Loss of *dilp2* does not influence the expression of known FOXO target genes and interactions with *dilp1* do not modify this result (Post et al., 2019). Flies lacking IPCs or with loss of dilps 2, 3, and 5 exhibit an abnormal response to diet manipulation (Grönke et al., 2010; Broughton et al., 2010), although *dFOXO* mutation leaves the response largely intact (Giannakou et al., 2008). Furthermore, loss of *dFOXO* only partially rescues longevity benefits of *chico* mutants (Yamamoto and Tatar, 2011).

Another nutrient-sensing pathway that acts cell non-autonomously in flies, like worms, is AMPK. Neuronal or intestinal activation of AMPK or Atg1 induces autophagy in both the brain and gut to slow organismal aging and improves numerous healthspan measures (Ulgherait et al., 2014). Dilps are implicated in mediating the inter-tissue response from the nervous system to the intestines and vice versa (Ulgherait et al., 2014). It is interesting that AMPK activation from several tissues causes metabolic remodeling across the whole-body. These data point to a feed-forward mechanism where signaling events occur bi-directionally to modulate the fly response to successfully survive stressful stimuli.

### Proteostasis signaling pathways

When it was first discovered that knocking-down mitochondrial electron transport chain components in nematodes extends lifespan, it seemed unlikely this phenomenon would be conserved in higher systems. Surprisingly, research using flies and mice (later discussed in section Mammals) points to a significant role for mitochondrial function in organismal lifespan. Global knockdown of ETC components in complexes I, III, IV, and V extends fly lifespan, but does not inhibit ETC complex formation or ATP production (Copeland et al., 2009). Furthermore, knockdown of complexes I and IV in neurons alone is sufficient to extend lifespan. This led researchers to ask how knockdown of the ETC extends lifespan. Follow-up studies show that knock-down of ETC complex I using ND75 RNAi in muscle tissue increases reactive oxygen species (ROS) and activates the mito-UPR and ImpL2 (insulin/IGF binding protein) (Owusu-Ansah et al., 2013). Subsequently, upregulation of mito-UPR target genes preserve muscle function while ImpL2 signals to the brain and fat body to decrease global insulin signaling. It is likely both pathways contribute to the longevity phenotype of decreased respiration chain expression, and there are data suggesting that ImpL2 increases lysosomal biogenesis and that autophagy genes are necessary for ND75 knock-down animals to live long. Another datapoint that suggests conservation of this pathway is that knock-down of ETC ND75 in complex I results in smaller flies, and a similar phenotype is documented in the nematode ortholog *isp-1* mutants (Feng et al., 2001).

Modifying the mitochondrial proton gradient by expressing human uncoupling proteins (hUCPs) modulates fly lifespan. Interestingly, the context of when and where the hUCPs are expressed play a critical role in health and longevity outcomes. hUCP2 targeted to the neurons increases health and longevity while increasing the rate of glycolysis and decreasing ROS production and oxidative damage (Fridell et al., 2005). Similarly, moderate pan-neuronal overexpression of hUCP3 leads to a modest lifespan extension exclusively in male flies (Humphrey et al., 2009). However, use of a stronger driver that targets hUCP3 pan-neuronally or to the median neurosecretory cells (mNSC) significantly decreases their lifespan. These data suggest that lowering uncoupling mitochondria by high expression of hUCP3 alters mNSC function in a way that increases DILP levels in fly heads and leads to a concomitant decrease in lifespan. Much is left to be done to fully understand the relationship between modulating mitochondria ROS levels and lifespan.

An important and robust area of research is focused on muscle maintenance with age. While slightly tangential to this review’s primary focus on longevity outcomes, the loss of muscle mass often precedes other age-related phenotypes like risks of falling. Throughout life, a fly’s muscles accumulate protein aggregates that impair function. Maintenance of proteostasis is enhanced in long-lived animals through elevated activity of FOXO target genes like 4E-BP that increase lysosome/autophagy functions (Demontis and Perrimon, 2010). Interestingly, FOXO signaling through 4E-BP activity in muscle decreases feeding behavior and the release of insulins that delay the age-related accumulation of protein aggregates in other tissues. This result suggests bi-directional cell non-autonomous signaling across tissues with a yet to be discovered signal (Demontis and Perrimon, 2010).

**Figure 5.**
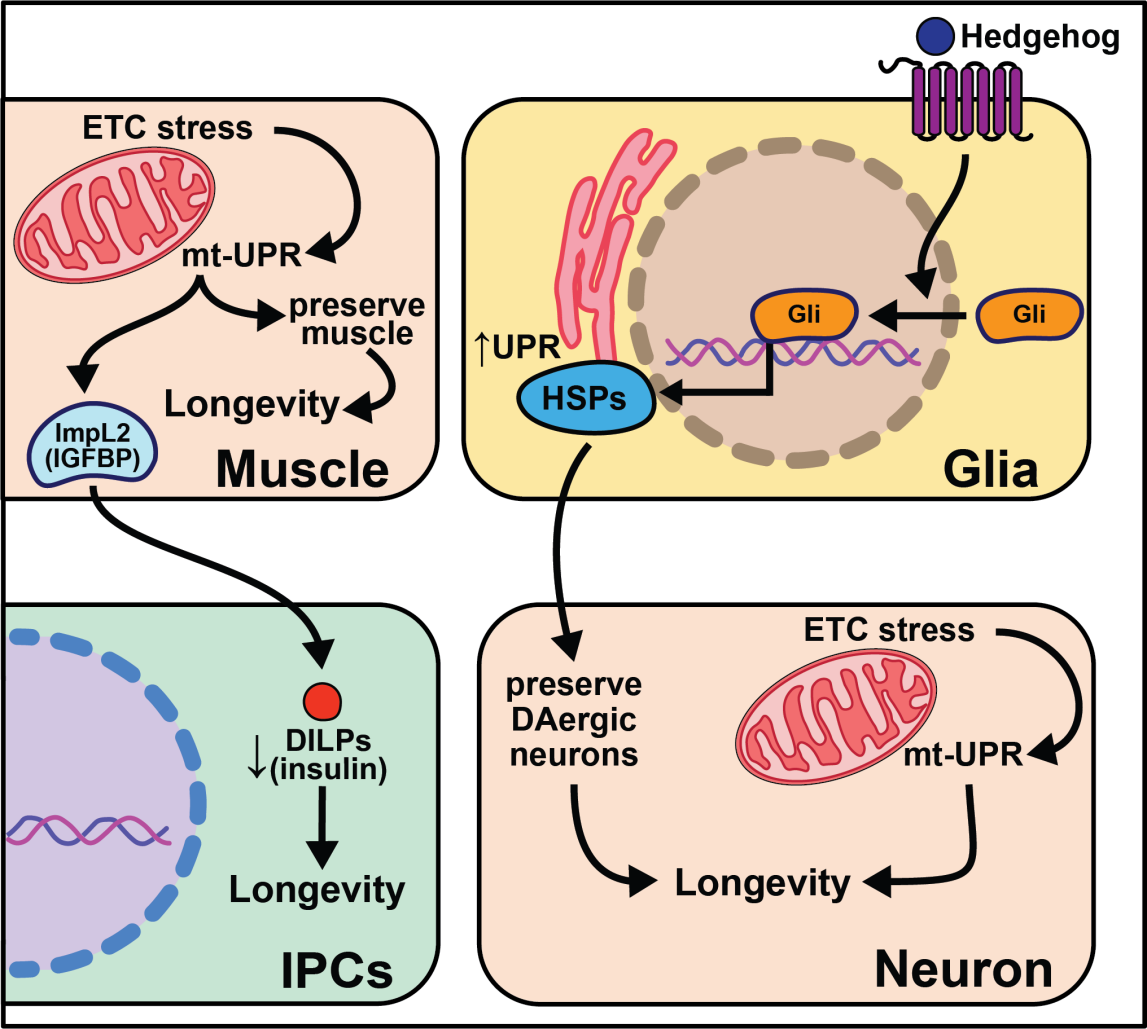
Summary of the role of proteostasis in cell non-autonomous modulation of longevity in *Drosophila melanogaster*. Mammalian orthologs are listed in parentheses. If there are no parentheses, the name is shared across taxa.

Overexpression of the gene hedgehog (*hh*), the Hedgehog signaling pathway ligand in *Drosophila,* extends lifespan, while disrupting this pathway shortens lifespan and decreases the number of dopaminergic neurons (Rallis et al., 2020). While overexpression of hedgehog signaling components in neurons has little effect on lifespan, overexpression in glia cells is sufficient to extend it. This work parallels nicely with work in *C. elegans* described above in which glial cells modulate aging through the UPR (Frakes et al., 2020). Overexpressing *hsp69* and *hsp40* in glia is sufficient to rescue lifespan in *hh* signaling mutants. Overexpression of smoothened (*smo)* and *hsp68* in glia partially rescues the lifespan shortening effects of expressing human Αβ42 plaques in *Drosophila* glia. Hh signaling may increase chaperone protein expression in adult glia, which act to maintain the integrity of dopaminergic neurons, leading to increased longevity (Rallis et al., 2020). While such data are compelling, it remains unclear how these neurons contribute to the lifespan extension of enhanced hedgehog signaling. It is also interesting to consider the conserved role glia cells play in neurotransmission, cell non-autonomous signaling, and longevity (Figure 5).

### Perception of external stimuli

**Figure 6.**
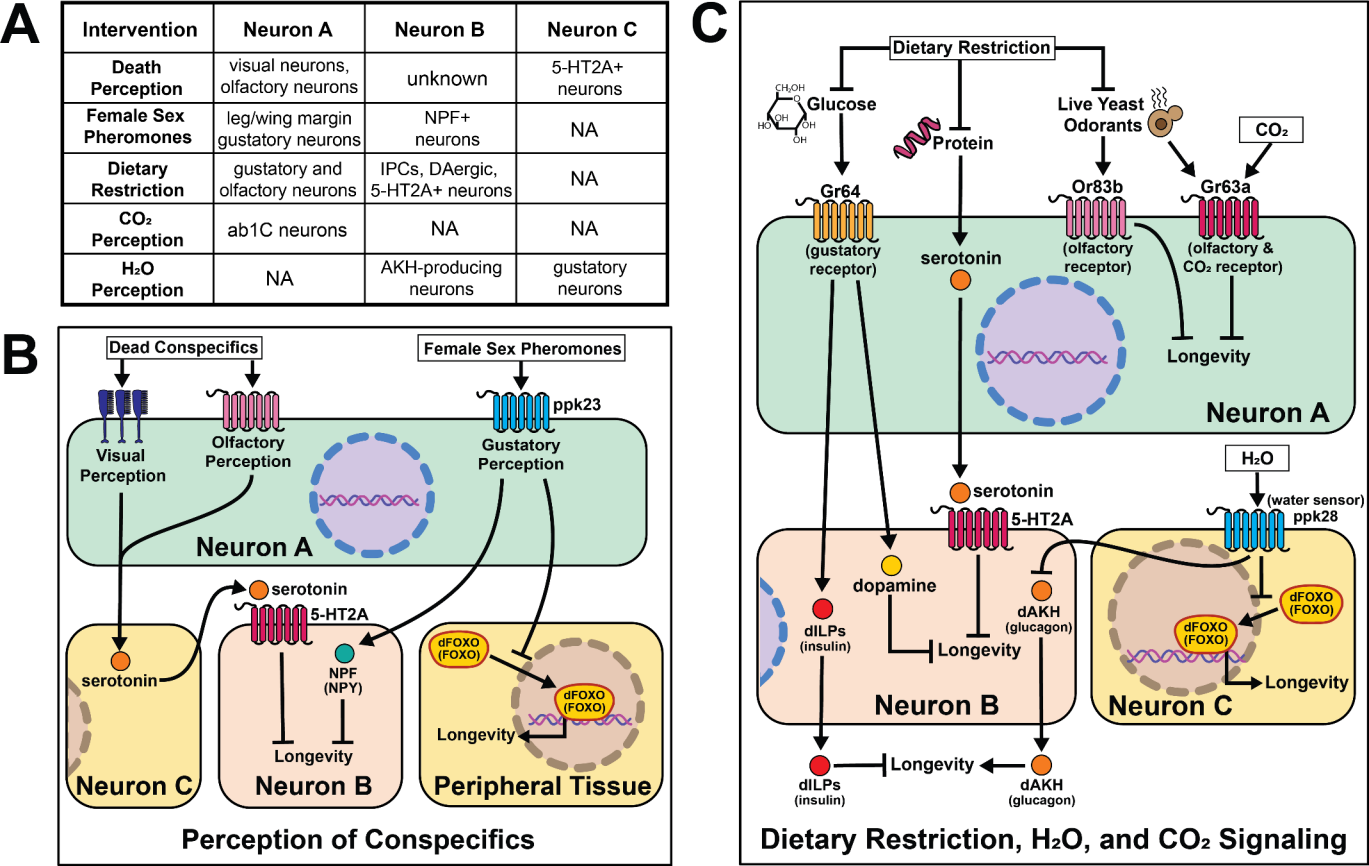
Summary of the role of perception on cell non-autonomous modulation of longevity in *Drosophila melanogaster.* Mammalian orthologs are listed in parentheses. If there are no parentheses, the name is shared across taxa.

Research in *Drosophila* has shepherded a significant expansion in our understanding of the effects of sensory perception on aging. Sensory inputs that relate information about nutrition, conspecifics, and danger rapidly initiate changes in fly physiology and patterns of aging, often within a few days. It is known, for example, that a restricted set of olfactory and gustatory neurons influence aging by either promoting or limiting lifespan, fat deposition, or general vigor in old age. Sensory perception of specific sugars and amino acids, as well as social signals such as the health of conspecifics or availability of potential mates, is also important. Conserved neuropeptides and functionally defined neuronal populations, some associated with psychological conditions such as reward and hunger, are involved in mediating these effects through new candidate cell-nonautonomous aging mechanisms (Figure 6).

Much of this work is centered around the effects of food perception. Exposure of flies to the odor of an important food source (live yeast) as well as knock-out of a critical co-factor for normal olfactory function (*Or83b*/*Orco*) established food perception is sufficient to partially reverse DR-mediated longevity (Libert et al., 2007). Loss of Orco function significantly increases fly lifespan. These effects are independent of food intake, activity, respiration, or early-life reproduction, suggesting a direct effect of sensory perception. Mutation of the water sensor, *ppk28*, extends *Drosophila* lifespan by up to 40%, and this effect requires AKH receptor (Waterson et al., 2014). AKH protein levels are higher in *ppk28* mutant animals, and activation of *Akh*-expressing neurons is sufficient to recapitulate the effects of loss of *ppk28* on lifespan. Gustatory perception is necessary for normal stress resistance and lifespan in a low-glucose environment (Linford et al., 2015). Loss of sweet taste receptor *Gr64* produces a sleep-impairment phenotype that is phenocopied by blocking dopamine neurotransmission, and taste-blind flies lived longer than control flies, despite eating more (Ostojic et al., 2014). Loss of the *Drosophila* trehalose receptor, *Gr5a*, significantly decreases lifespan without altering feeding (Waterson et al., 2015), establishing that taste inputs can modulate lifespan in both directions. Similar to some methods of diet restriction in *C. elegans*, loss of labellar taste bristles requires insulin signaling to extend lifespan (Ostojic et al., 2014).

In *Drosophila,* the effects of dietary restriction on aging are predominantly influenced by dietary composition (mainly protein content) rather than the overall caloric content of the food (Solon-Biet et al., 2014; Mair et al., 2005; Skorupa et al., 2008; Simpson and Raubenheimer, 2007). Flies exposed to restriction of essential amino acids behaviorally switch from a diet comprised primarily of sugar to one primarily of protein (Steck et al., 2018). This behavioral switch in feeding preference requires both serotonin signaling through the 5-HT2A receptor and plasticity of a dopaminergic circuit (Ro et al., 2016; Liu et al., 2017). When the two primary macronutrients in the diet, sugar and protein, are presented separately to flies so that they behaviorally construct the composition of their own diet, they live shorter than when presented with a single, complete diet (Ro et al., 2016) an effect that also requires serotonin signaling through the serotonin receptor 5-HT2A. This suggests that protein sensing may be mediating this effect. Finally, serotonin, 5-HT2A, and the solute carrier 7-family amino acid transporter, JHL-21, modulate diet-dependent aging by ascribing value to ingested protein. Interestingly, *JhI-21* is expressed in the reproductive tissues, which are a primary consumer of dietary protein, suggesting an inter-tissue communication.

Outside of a flies’ ability to assess food quality, several other sensory cues rely on neuronal signaling to modulate lifespan. Exposure to female sex pheromones in the absence of mating causes rapid and reversible declines in fat stores, stress resistance and longevity in male flies (Gendron et al., 2014). Changes in metabolism and lifespan require taste perception through the gustatory receptor, *ppk23,* as well as neuronal signaling involving the conserved neuropeptide NPF/NPY and FOXO. These effects are partially reversed by copulation, suggesting that survival costs of reproduction in male flies are controlled by neural circuits through which reproductive expectation dictates costly precopulatory investment in reproductive success. Related circuits that perceive reproductive reward ameliorate the consequences of this investment if mating is achieved (Harvanek et al., 2017). Notably, mating decreases both worm and fly lifespan, it is worthwhile to ponder why perception of imminent mating without achieving it causes greater physiological harm to a flies’ health. Perhaps upon completing a satisfying activity that is evolutionarily beneficial, like eating or mating, specific neural circuits and signaling peptides reinforce these behaviors, and without these signals only detrimental effects remain.

Cues that putatively signal danger are also important. *Drosophila* can visually perceive dead conspecifics in their environment and this perceptive experience induces both short- and long-term effects on health and longevity. Exposure to dead flies decreases resistance to starvation, depletes lipid storage, and shortens lifespan (Chakraborty et al., 2019). As with protein perception, serotonin signaling via receptor 5-HT2A is required for death perception to influence lifespan. With the advent of new technologies, it would be interesting to test whether the same or different neuron populations require 5-HT2A receptors to influence death and/or food perception. *Gr63a* encodes for one of two proteins which make up a CO_2_ receptor, and at low concentrations CO_2_ is a known alarm cue (Suh et al., 2007). Flies with a loss-of-function mutation in *Gr63a* are long-lived and are additive with ab1C neuronal ablation suggesting DR and alarm-sensing act through distinct pathways to influence lifespan (Poon et al., 2010).

## Mammals

**Figure 7.**
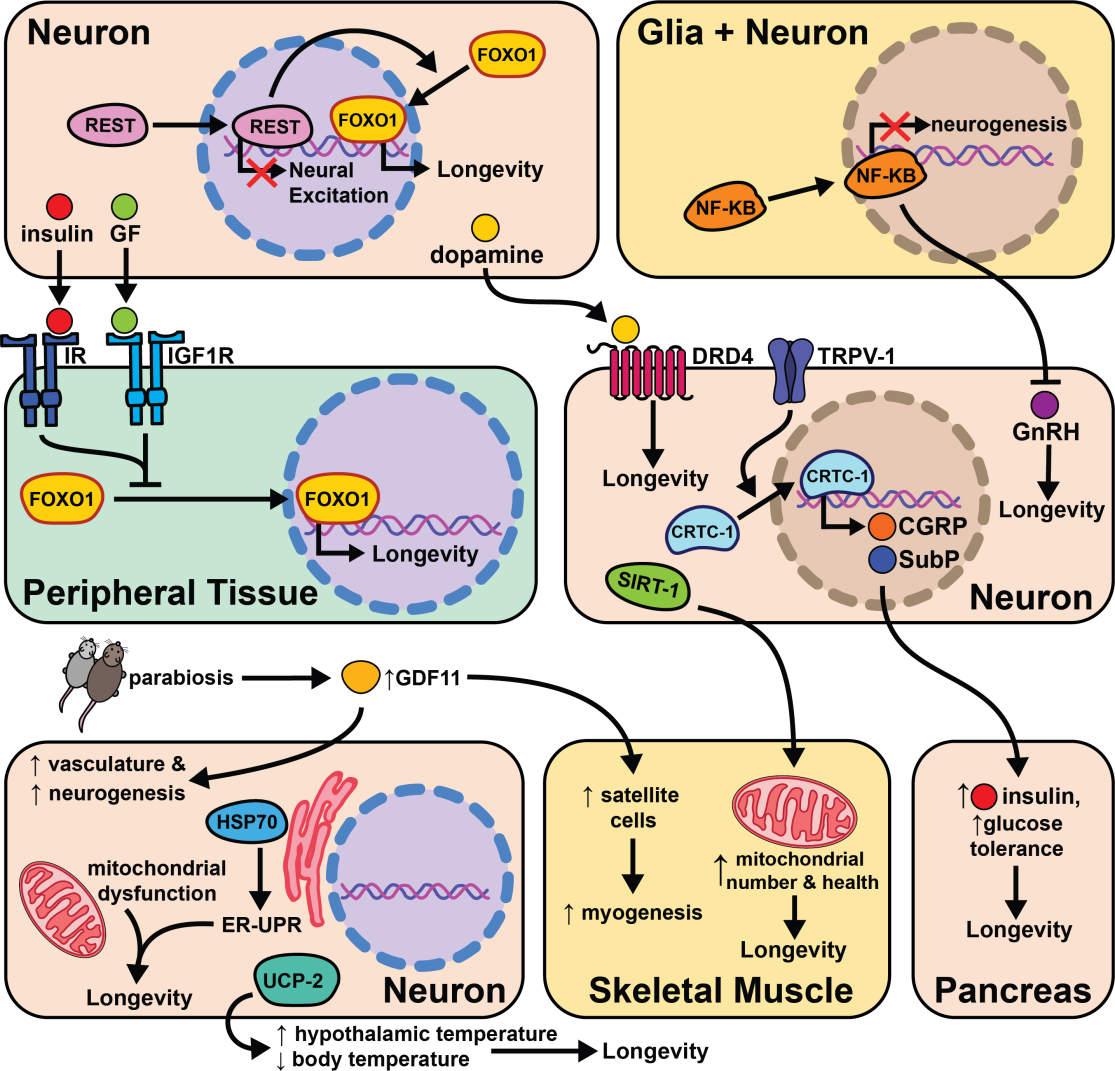
The intersection of cell non-autonomous signaling and aging in mammals.

Fewer studies have explored the effects of aging and cell non-autonomous signaling in mammals due to the extended amount of time and effort needed to perform these experiments. Despite these considerations, a growing body of literature suggests the types of signaling events seen in invertebrates are conserved from worms to mice. These initial findings portend an increase in the number of studies investigating the effects of the nervous system on aging. In this section, we will discuss the individual studies performed in mice and compelling evidence that suggests this phenomenon may be conserved in humans (Figure 7).

Similar to the effects observed in worms and flies, reduced insulin and growth hormone signaling also significantly increase mouse lifespan (Brown-Borg et al., 1996). Ames dwarf mice, and the similar Snell dwarf mice, each have a single-gene mutation, Prop1 and Pit1, respectively, that impairs anterior pituitary development leading to decreased growth hormone, thyroid stimulating hormone, and prolactin levels. These mice live 40% longer than controls and have decreased plasma IGF-1 (Flurkey et al., 2002). However, dwarf mice do become obese in old age, showing that pituitary modulation of aging is independent of body weight regulation. Interestingly, grafting a control pituitary gland in adulthood does not rescue control lifespan in Snell dwarf mice (Flurkey et al., 2001). The mechanism for longevity likely involves IGF-1, because similar results have been obtained using the knockout for growth-hormone receptor binding protein (GHR-KO) (Coschigano et al., 2003), a heterozygous null mutation of the IGF-I receptor (Holzenberger et al., 2003), and the knockout of growth hormone releasing hormone (lit/lit mice, mutant for Ghrhr) (Flurkey et al., 2001). Similarly, partial loss of function of IGF-1R in neurons during development leads to reduced growth and lifespan extension (Kappeler et al., 2008). These mice gain slightly more weight with age than WT controls and have higher levels of subcutaneous adipose tissue, higher circulating leptin, and higher circulating lipids. These studies show decreased insulin signaling can alter global metabolism and extend lifespan in mammals. While they do not directly test the effects of other types of neural signaling, it does confirm that insulin signaling influences lifespan in mice.

Related work in humans suggests insulin regulation, sensitivity, and neuronal excitability all positively correlate with longevity. A transcriptome analysis of cerebral cortex tissue compared >85-year-old humans to the <80-year-old group. One transcriptional repressor, REST, negatively regulates neural excitation and FOXO1 expression, and its expression positively correlates with longevity (Zullo et al., 2019). In *C. elegans* REST orthologues *spr-3* and *spr-4* are required for *daf-2*/IGFR knockout lifespan extension. The signaling mechanisms between neuronal REST activity and FOXO1 expression in peripheral tissues are not known, but these data provide ample evidence of conserved cell non-autonomous modulation of long-term health.

Similarly, genetic studies of centenarians have identified the locus encoding for tyrosine hydroxylase (TH), insulin (INS), and Insulin Growth Factor 2 (IGF2) as correlative with longevity (De Luca et al., 2001). TH is the rate-limiting enzyme responsible for producing the neurotransmitter dopamine (Nagatsu, 1995). This study looks more closely at the association of specific INS and IGF2 polymorphisms with longevity in humans. Polymorphisms in the subregion spanning TH and INS were significantly associated with lifespan in females, while polymorphisms in the region spanning TH and IGF2 were significant in males. These data support the role of insulin and dopamine signaling in human lifespan, and the gender difference observed may be explained by variations in metabolism between the sexes in old age. Supporting these findings, another research group genotyped a cohort of 90–109 year-olds and compared them to ancestry-matched younger people (ages 7–45). The 90+ cohort had a 66% higher incidence of a specific allele of the dopamine D4 receptor (DRD4 7R) which correlated with higher physical activity (Grady et al., 2013). Additionally, DRD4 knockout in mice leads to an ~8% decrease in lifespan. It’s unclear what changes in dopaminergic signaling occur in these two populations and whether the increased physical activity is directly linked with the DRD4 allele.

Mice heterozygous at the insulin receptor substrate 2 (Irs2) locus do not differ from their control counterparts in food intake or body weight, but have significantly increased insulin sensitivity and live ~25% longer (Taguchi et al., 2007). Knocking out Irs2 in neurons (bIrs2) is sufficient to phenocopy the lifespan extension in the global knockout, and leads to decreased mRNA expression of superoxide dismutase 2 (Sod2) and Foxo1. These results support previous hypotheses that reduced insulin signaling in neurons modulates lifespan by enhanced protection from oxidative stress. It will be interesting to test whether enhanced Irs2 expression in the nervous system shortens mice lifespan.

Recent studies address questions about initiation and duration of a longevity intervention. Most studies introduce dietary or therapeutic interventions while the mice are young adults (de Cabo et al., 2014). This method is not entirely translatable to humans as we would likely be middle-aged or elderly before adopting a pro-longevity treatment regimen. Many labs are beginning to address this concern by testing the effects of longevity treatments after reaching midlife (Harrison et al., 2009; Bitto et al., 2016). Applying these principles, supplementing middle-age and elderly mice with intranasally administered recombinant human Hsp70 extends lifespan by ~10% and improves learning and memory during old age (Bobkova et al., 2015). Interestingly, Hsp70-treated mice had higher neuronal density in the temporal cortex and the hippocampus and immunostaining of the cerebral cortex for ribosomal proteins reveals more accumulation of proteasomal subunits in the Hsp70-treated mice. This suggests that Hsp70 promotes proteasomal activity and can extend lifespan in mammals, presumably through both cell autonomous and cell non-autonomous mechanisms.

The hypothalamus is a key producer of neuropeptides and hormones. It is likely many signals from the hypothalamus are important to relay information from neurosignaling pathways to the rest of the body. Additionally, the hypothalamus is key to maintaining homeostasis in energy balance, blood pressure, oxygenation, body temperature, circadian rhythm, etc (Barbosa et al., 2017). Therefore, any perturbations in environment/environmental stressors are likely transmitted from sensory neurons to the hypothalamus. Overexpressing uncoupling protein 2 (UCP2) in mouse hypocretin neurons (Hcrt) increases body temperature specifically in the hypothalamus and leads to a decrease of 0.3–0.5°C in core body temperature (Conti et al., 2006). These transgenic Hcrt-UCP2 mice have the same calorie intake relative to WT controls, but live 12–20% longer (Conti et al., 2006). These data suggest that neuronal regulation of core body temperature influences lifespan independently of DR and supports cell non-autonomous modulation of aging in mice.

The significant role that the hypothalamus plays in cell non-autonomous modulation of aging is further supported by experiments modulating neuronal NF-κB levels in middle-aged mice. NF-κB is a well-studied transcription factor involved with inflammation and the immune response (Taniguchi and Karin, 2018). As aging is correlated with increased inflammation, this study asked whether changes in NF-κB expression with age lead to pro- or anti-aging phenotypes. Middle-aged mice with activated NF-κB in the hypothalamus have slightly shorter lifespan, whereas NF-κB inhibition extends lifespan by ~15% (Zhang et al., 2013). NF-κB inhibition improves maze-learning, muscle endurance, and bone mass while NF-κB knockout in hypothalamic microglia is sufficient to phenocopy the lifespan extension from neural inhibition of NF-κB. Both neuronal and glial hypothalamic NF-κB knockdown also lead to increased gonadotropin-releasing hormone (GnRH) mRNA expression in old mice. GnRH neurons are hypothalamic cells that regulate fertility through pulsatile GnRH release, and importantly, increases in GnRH expression or treatment of mice with GnRH injection correlates with improved neurogenesis and lifespan. The implication of this study is that increased inflammation from NF-κB expression during aging leads to loss of GnRH release and subsequent diminished health, and that restoring GnRH levels can reverse this effect. These data not only support a role for the hypothalamus in cell non-autonomous modulation of aging, but suggest a plausible signaling mechanism (GnRH release) for this role.

Recent work further explores the role of the brain in influencing health and longevity in mice. Brain-specific Sirt1 OE (BRASTO) mice live 10–15% longer than controls and have decreased cancer incidence (Satoh et al., 2013). Middle-aged (20 month) BRASTO mice also exhibit improved healthspan parameters when compared to their aged matched controls; they are more physically active, have higher core body temperature, consume more oxygen, and have more non-REM sleep. Similar traits are often correlated with increased quality of life in elderly humans (Rea et al., 2015; Spadafora et al., 1996; Simonsick et al., 2016). While the causative mechanisms behind the improved health and longevity of BRASTO mice are not fully characterized, they show an increase in skeletal muscle mitochondria and mitochondrial functional gene expression in addition to higher mRNA expression of markers of neuronal activity in hypothalamic neurons. Further studies will hopefully identify how SIRT1 expression in the brain changes downstream physiology to improve health and longevity. This will likely involve modulation of neuronal signaling, perhaps due to improved health of neurons in the brain. It will also be interesting to find whether and how these changes are distinct from and overlap with pathways such as insulin-like signaling, that also emanate from the brain. This study further supports the role of neurons, and hypothalamic neurons in particular, as key modulators of cell non-autonomous aging.

Cellular senescence is a process characterized by permanent cessation of cellular proliferation. There is a large body of evidence supporting the hypothesis that the proliferation of senescent cells throughout an animal’s lifetime will accelerate their aging via pro-inflammatory secreted compounds. Plasminogen activator inhibitor-1 (PAI-1), a neuronally expressed protein, regulates cellular senescence in mammals (Carmeliet et al., 1993; Eren et al., 2002). Klotho is an ‘aging-suppressor’ gene and klotho knockout mice exhibit an accelerated aging phenotype and increased plasma PAI-1 levels when compared to age-matched controls (Eren et al., 2014). Knocking out PAI-1 in klotho mutant mice reduces senescence, normalizes telomere length, preserves organ function, and completely rescues their lifespan. These mice are likely short-lived due to accumulation of pro-inflammatory molecules from the ‘senescence messaging secretome’ (SMS) that influences aging through cell non-autonomous signaling. In support of this hypothesis, there is mounting correlative data suggesting elevated PAI-1 levels in humans is strongly associated with aging disease states (Yamamoto et al., 2005). While these data are intriguing, more experiments are needed to test whether neuronal SMS mechanistically accelerates aging.

Cell non-autonomous signaling from senescent cells is another likely mechanism of influencing aging. Mammals accumulate senescent cells throughout life, and when chronically present, senescent cells exacerbate age-dependent tissue deterioration due to inflammatory signals dubbed the senescence-associated secretory phenotype (SASP) (Campisi and Robert, 2014). However, when transiently present, senescent cells can promote healthy outcomes like optimal wound healing (Demaria et al., 2014). While the local effects of these signals have been well-studied, their systemic effects remain unclear (Campisi, 2013). Much work is left to parse out the efficacious effects of senescent cells from SASP, but it is compelling that many anti-aging therapeutics shown to extend mouse lifespan seem to target and kill senescent cells (Campisi et al., 2019). Whether the lack of these senescent cells is responsible for the pro-longevity effects from these drugs remains unclear. Senolytic compounds have recently entered clinical trials to test their efficacy in treating age-associated diseases (Kulkarni et al., 2020) and represent a local and possibly systemic mechanism for cell non-autonomous modulation of aging.

The process of identifying pro- and anti-aging signaling events such as SASP is accelerated by the use of heterochronic (differently aged) animals sharing a circulatory system either through parabiosis or serum transfer. Incredibly, circulating factors in the blood of young mice can restore cellular function in older mice. Specifically, old mice exposed to young serum show enhanced Notch signaling in satellite cells, increased hepatocyte proliferation, enhanced neurogenesis, decreased incidence of cardiac hypertrophy, and a reduced SASP response (Conboy et al., 2005; Loffredo et al., 2013; Katsimpardi et al., 2014; Rebo et al., 2016; Yousefzadeh et al., 2020). Follow-up studies show the TGF-β superfamily member protein GDF11 decreases with age and restoring its levels-alone is sufficient to reverse age-related dysfunction in the skeletal system (Sinha et al., 2014) as well as restoration of the neurogenic niche (Katsimpardi et al., 2014). Despite the observed benefits of young serum in older mice, an initial study found that young plasma is not able to extend lifespan in aged mice (Shytikov et al., 2014). Together, these results provide the basis for ongoing work identifying the mechanisms of these observations with the hope of showing that defined ‘youth factors’ can improve human healthspan, lifespan, or both. These data highlight the significant role circulating proteins and secreted compounds likely play in modulating aging cell non-autonomously.

Labs with a previous invertebrate focus have begun to explore whether cell non-autonomous signaling pathways are conserved from invertebrates to mammals. To that end, globally knocking out the capsaicin receptor TRPV1 in mice causes no change in body weight or circulating growth hormone (GH)/IGF-1, but increases metabolic activity and energy expenditure and increases lifespan by 10–20% (Riera et al., 2014). In worms, knocking out the TRPV-1 orthologues, *osm-9* and *ocr-2*, requires the transcription factor CRTC-1 to influence lifespan. In mouse dorsal root ganglion (DRG) primary neuron cultures, TRPV-1 is also necessary for nuclear translocation of CRTC-1. CRTC-1 interacts with CREB in DRG neurons to regulate secretion of the neuropeptides calcitonin gene-related peptide (CGRP) and substance P at pancreatic beta cells. The model suggests TRPV-1 knockout mice have reduced CRTC-1 activity which reduces CGRP expression and increases pancreatic insulin secretion leading to better glucose tolerance and longevity. It is likely this type of translational study will become more common in the future, establishing which cell non-autonomous pathways of aging are conserved from invertebrates to mammals.

Together, mammalian aging studies show a clear role for cell non-autonomous signaling, but with less detail than in invertebrate organisms. Similar to invertebrates, insulin-like signaling represents the best studied pathway, but additional details are continuously emerging. Whether emanating from whole brain (SIRT1), regions of the brain (e.g. hypothalamic NF-κB and UCP), or from individual tissues throughout the body (SASP, parabiosis), cell non-autonomous modification of systemic aging plays a role in mammals. For many of these pathways, the field is very new and more exciting data will likely come in the future.

## Emerging concepts

**Figure 8.**
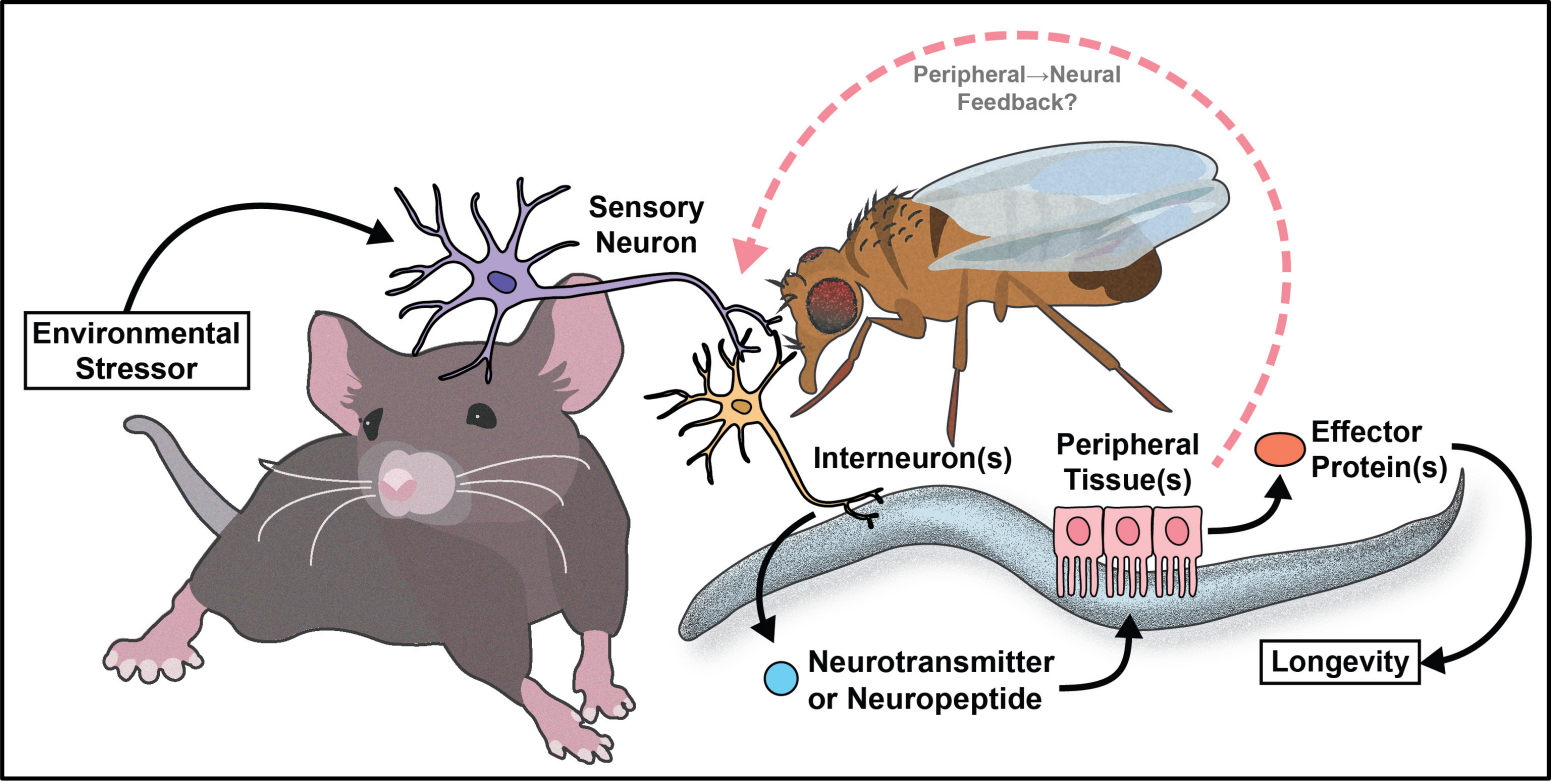
Summary model.

The work reviewed here (summarized in Figure 8) represents a small sliver of the extensive discoveries the aging field has made in three decades. Despite cell non-autonomous signaling being a relatively new concept to the field, the critical role the nervous system plays in promoting healthy aging is well established. Moreover, by understanding how key signaling tissues evaluate and appropriately integrate large amounts of internal (energy stores and the allocation of resources) and external (availability of food/sexual partners and quality) stimuli, we can target the decision-making processes to mimic pro-longevity stimuli.

Despite the clear role that cell signaling plays in maintaining proteostasis, oxygen homeostasis, food regulation, and overall physiology, there is much we do not know about these areas. All fields, but invertebrate research in particular, could follow the lead of the behavior field, where there is convincing data establishing neural circuits and signaling molecules that regulate aspects of behavior. The aging field has occasionally completed similar work, but much less is understood about how cells recognize and relay signals about different types of internal and external environmental stimuli. This is to be expected, as behavioral outcomes are often measurable in seconds or minutes, whereas long-term health and lifespans require weeks or months (for invertebrates) or years (for mammals). Due to time constraints, this is an area where invertebrate research will likely need to lead, but once established, the translatability of the networks should be measured. A subset of invertebrate studies do establish key aspects of these circuits, and if more effort was put into this endeavor, we may also find whether behavior and long-term health interact or are controlled by entirely distinct pathways.

With the establishment of cell non-autonomous regulation of aging in multiple pathways and organisms, there is immense therapeutic potential for this area going forward. Most therapeutics logically target the tissues where physiological change is important, while understanding signaling networks provides a unique opportunity to use the natural signaling network to ‘trick’ key tissues into improving long-term health. This will not necessarily be easy, as targeting neural circuits using broad drugs (e.g. SSRIs) often have pleiotropic effects, but the better we understand the signaling networks the more specifically we could, in theory, mimic the signal(s). Using a signaling approach to anti-aging therapeutics would allow for induction of hormetic effects without the need for an acute stress, and has great potential to mimic well-established longevity interventions such as dietary restriction. It will be of paramount importance, as the field continues to mature, that we test the conservation of these networks from simple worms to complex mammals. It is probable, however, that like the first discovered cell non-autonomous network to influence aging (insulin-like signaling), other pathways will be partially or largely conserved.

To that end, drug screens in *C. elegans* for pro-longevity therapeutics have found neuromodulators that extend lifespan. More specifically, it is intriguing that serotonin antagonism can extend adult neuroplasticity and lifespan (Petrascheck et al., 2009; Bazopoulou et al., 2017). It is interesting that antagonizing serotonin signaling or other reward circuitry can lead to the same physiological changes that occur under hormetic stress. Using drug combinations to simultaneously target multiple aging pathways has also shown promise in *C. elegans* and *D. melanogaster*. For example, Admasu *et. al* identified two drug combinations that synergistically improve lifespan and healthspan. The synergistic effects of both drug combinations required TGF-β signaling and increased levels of monounsaturated fatty acids (Admasu et al., 2018). Combining multiple anti-aging pharmaceuticals in flies has also proved efficacious. Simultaneous inhibition of mitogen-activated protein kinase kinase, mTOR complex 1, and glycogen synthase kinase-3 acted additively to increase *Drosophila* lifespan by 48% (Castillo-Quan et al., 2019). These data suggest it may be possible to co-opt these pathways with small molecules to slow mammalian aging. This is crucial since it is likely humans will not change their environment (e.g. dietary restriction) in spite of potential benefits. By better understanding the molecular and signaling mechanisms of these pathways, these processes can be targeted directly, attaining benefits to human health while circumventing the challenges of implementing population-scale environmental perturbations.

Much of the work reviewed here investigates how the nervous system communicates with peripheral tissues to influence aging. This can be thought of as canonical cell non-autonomous signaling. However, recent data from invertebrates supports the idea of non-canonical cell non-autonomous mechanisms where the peripheral tissues use a retrograde signal back to the nervous system to maintain or further modify physiology (Minniti et al., 2019; Bouagnon et al., 2019; Demontis and Perrimon, 2010; Ulgherait et al., 2014). This type of signaling is logical, as organisms require feedback from individual tissues to monitor homeostasis, but the role of retrograde signaling in regulation of aging is not well understood. This concept presents an interesting case for future studies to investigate the circuitry events that occur from the downstream metabolic tissues back to the nervous system. It also provides an opportunity to better understand how cells at the interface of forward and retrograde signaling (i.e. the hypothalamus) make decisions that affect both upstream and downstream physiology.

Another area that will be crucial in future studies will be the identification of epigenetic regulation of these cell non-autonomous networks. While sentinel-like cells such as neurons signal to peripheral tissues to modify stress resistance and longevity, how these pathways maintain their benefits over time is not well understood. Studies show that just a day of hypoxia (Mehta et al., 2009), for example, can extend lifespan in a worm, but whether that is just due to the persistent benefits of physiological changes made during that day or due to lasting epigenetic changes in peripheral tissues is an open question. The answer will give clues as to whether we could develop therapeutics that are only taken intermittently or whether more continuous treatment is necessary to extend healthspan. Additionally, studies in this area could separate how organisms respond to acute and chronic stress, and whether a series of acute activations of stress responses bring about long-term benefits.

Together, cell non-autonomous regulation of aging represents an exciting area of study that is well-established with many exciting but open questions. The future of this area has great potential to both improve our understanding of the aging process and lead to useful therapeutic advances to improve human health.
